# Fluid overload and changes in serum creatinine after cardiac surgery: predictors of mortality and longer intensive care stay. A prospective cohort study

**DOI:** 10.1186/cc11368

**Published:** 2012-05-31

**Authors:** Anna Stein, Lucas Vieira de Souza, Cassian Rodrigues Belettini, Willian Roberto Menegazzo, Júlio Rosales Viégas, Edemar Manuel Costa Pereira, Renato Eick, Lilian Araújo, Fernanda Consolim-Colombo, Maria Cláudia Irigoyen

**Affiliations:** 1 Intensive Care Unit, Instituto de Cardiologia do Rio Grande do Sul/Fundação Universitária de Cardiologia (IC/FUC), Princesa Isabel av 395, Porto Alegre, 90620-001, Brazil; 2 Nephrology Division, Department of Medicine, School of Medicine, Federal University of Sao Paulo, Sena Madureira st 1500, São Paulo, 04021-001, Brazil; 3 Hypertension Unit, Heart Institute (INCOR), Dr. Enéas de Carvalho Aguiar av 44, São Paulo, 05403-900, Brazil; 4 Medical Pos Graduate Program, Nove de Julho University, Vergueiro st 249, São Paulo, 01504-001, Brazil

## Abstract

**Introduction:**

Fluid overload is a clinical problem frequently related to cardiac and renal dysfunction. The aim of this study was to evaluate fluid overload and changes in serum creatinine as predictors of cardiovascular mortality and morbidity after cardiac surgery.

**Methods:**

Patients submitted to heart surgery were prospectively enrolled in this study from September 2010 through August 2011. Clinical and laboratory data were collected from each patient at preoperative and trans-operative moments and fluid overload and creatinine levels were recorded daily after cardiac surgery during their ICU stay. Fluid overload was calculated according to the following formula: (Sum of daily fluid received (L) - total amount of fluid eliminated (L)/preoperative weight (kg) × 100). Preoperative demographic and risk indicators, intra-operative parameters and postoperative information were obtained from medical records. Patients were monitored from surgery until death or discharge from the ICU. We also evaluated the survival status at discharge from the ICU and the length of ICU stay (days) of each patient.

**Results:**

A total of 502 patients were enrolled in this study. Both fluid overload and changes in serum creatinine correlated with mortality (odds ratio (OR) 1.59; confidence interval (CI): 95% 1.18 to 2.14, *P *= 0.002 and OR 2.91; CI: 95% 1.92 to 4.40, *P *<0.001, respectively). Fluid overload played a more important role in the length of intensive care stay than changes in serum creatinine. Fluid overload (%): b coefficient = 0.17; beta coefficient = 0.55, *P *<0.001); change in creatinine (mg/dL): b coefficient = 0.01; beta coefficient = 0.11, *P *= 0.003).

**Conclusions:**

Although both fluid overload and changes in serum creatinine are prognostic markers after cardiac surgery, it seems that progressive fluid overload may be an earlier and more sensitive marker of renal dysfunction affecting heart function and, as such, it would allow earlier intervention and more effective control in post cardiac surgery patients.

## Introduction

Cardiac surgery is the surgical procedure most frequently associated with acute kidney injury (AKI) [1]. Kidney dysfunction during the perioperative period has also been associated with increased length of hospital stay [2] and with a mortality rate as high as 50% [3], regardless of the underlying disease [4]. Therefore, research has been carried out with various biomarkers in order to determine their prognostic value.

Creatinine is the most widely used marker of kidney function in patients undergoing cardiac surgery. According to Lassnigg, a small increase in serum creatinine (0 to 0.5 mg/dL) has been associated with 30-day mortality [5]. On the other hand, fluid overload has also been linked with worse prognosis in several situations, including heart failure [6,7]. Due to this close interaction, fluid overload has been identified as a new biomarker of heart and renal function [8]. Cardio renal syndrome combines kidney dysfunction and heart failure in many clinical conditions. In kidney and in heart dysfunction, fluid overload is generally regarded as an important clinical condition [9,10]. Fluid overload exerts greater venous pressure on the kidney, reducing kidney perfusion and glomerular filtration [11]. However, fluid overload often remains symptomless for several days until clinical symptoms set in, when treatment is usually initiated [12].

Classically, creatinine has been used to identify kidney dysfunction; however, serum creatinine may remain within the normal range until about half of the kidney function is lost [13]. Hence, clinical diagnosis of kidney dysfunction might be dangerously delayed, as might renal and heart protective procedures. Brain Natriuretic Peptide (BNP) and pro BNP are widely used biomarkers for heart failure, but when the glomerular filtration rate is less than 60 ml/minute their levels become very high, reducing their potential diagnostic accuracy [14]. However, there is no evidence of the relative importance of fluid overload detection and/or changes in serum creatinine in terms of their relative sensitivity as markers of the risk of early mortality in the postoperative period following cardiac surgery.

## Materials and methods

### Study design

We conducted a cohort study. This study was approved by the Ethics Committees of the Instituto de Cardiologia do Rio Grande do Sul/Fundação Universitária de Cardiologia (IC/FUC), Porto Alegre, RS, Brazil and Universidade Federal de São Paulo (UNIFESP), São Paulo, SP, Brazil, and registered under the numbers 4560/10 and 1781/107, respectively. Data were collected anonymously following the clinical routine, with a waiver for informed consent. We conducted a one year cohort prospective study of all patients from the Postoperative ICU who underwent surgery involving cardiopulmonary bypass (CPB) in a tertiary cardiac referral hospital, Instituto de Cardiologia do Rio Grande do Sul/Fundação Universitária de Cardiologia (IC/FUC), beginning September 2010. Inclusion criteria were as follows: adult patients (>18 years old) submitted to the following elective surgical procedures: myocardial revascularization surgery, valve replacement surgery, valve replacement surgery plus myocardial revascularization, atrial septoplasty and ventricular septoplasty. The exclusion criteria were as follows: length of stay in the Postoperative ICU less than 24 hours, loss to follow-up and patients submitted to surgical re-intervention during the same hospital stay.

The hypothesis of this study was that fluid overload is independently related to mortality and length of stay in the ICU following cardiac surgery. Thus, the purpose of this study was to evaluate the effects of fluid overload and serum changes in serum creatinine on death and length of ICU stay and mortality in patients who underwent heart surgery, regardless of other perioperative risk factors.

We followed the STROBE statement of observational studies.

### Data collection

#### Study population methodology

Preoperative demographic and risk indicators, intra-operative parameters and postoperative information were obtained from medical records. The preoperative variables were age (in full years), gender, weight (kg), serum creatinine (in mg/dL), EuroSCORE risk and related diseases. Serum creatinine was measured using the automated colorimetric method (Roche® Mannheim, Germany), reference values from 0.30 to 1.30 mg/dL. At our hospital, the routine creatinine analysis variation was 1.4%. Cardiac function was quantified using the ejection fraction (%) obtained by the echocardiogram. During the intra-operative period, fluid balance (fluid administered - fluid excreted - in mL) and CPB time (in minutes) were evaluated.

During postoperative follow up all available liquid intake and output data from surgery day until ICU discharge or death were included. Fluid balance (fluid administered - fluid excreted - in mL) and changes in serum creatinine were evaluated daily. Fluid management and all other interventions were determined by attending physicians and were not influenced by the study researchers. Patients were monitored from surgery until discharge or death during ICU stay period. We also evaluated the survival status and the total length of ICU stay (in days) of each patient.

To assess the impact of fluid accumulation and acute kidney dysfunction on mortality and length of ICU stay, percentage fluid accumulation and changes in serum creatinine were measured and analyzed.

For fluid accumulation, we considered the whole length of the ICU stay, including the day of surgery. We measured the 24-hour totals of fluid intake and output daily, including the trans-operative surgical period. In order to calculate daily fluid balance, the following formula was applied daily: Total fluid received (L) - Total amount of fluid eliminated (L).

In order to quantify fluid accumulation, the following formula was applied: Daily fluid accumulation sum = [Total quantity of fluid received (L) - total amount of fluid eliminated (L)]/Preoperative weight (kg) × 100. This percentage of fluid accumulation was calculated according to procedures found in previous studies [15,16]. We used the term 'percentage of fluid accumulation' to define the percentage of cumulative fluid adjusted for body weight. We define fluid overload as ≥10% of fluid accumulation, following a previously applied classification [17,18]. To measure changes in serum creatinine, for each patient we calculated the difference between the highest serum creatinine value observed in ICU and preoperative creatinine. To identify if the fluid accumulation might interfere with the dilution value of changes in creatinine, we used a correction factor, according to Macedo [19]: Adjusted creatinine = serum creatinine × correction factor.

$$\text{Correction}\;\text{factor}=\left(\text{preoperative}\;\text{weight}\left(\text{kg}\right)\times 0.\text{6 + }\Sigma \left(\text{daily cumulative}\;\text{fluid}\;\text{balance (L}\right)\right))/\text{pre}\;\text{operative weight}\;\times 0.6$$

We also evaluated age, EuroSCORE risk, preoperative creatinine, stratified changes in creatinine (≤0.3 mg/dL, 0.3 to 0.6mg/dL and ≥0.6 mg/dL), stratified volume accumulation (≤5%, 5% to 10%, ≥10%) and CPB time as potential confounding factors. We considered infection, pulmonary edema, bleeding, cardiac arrhythmia and death as combined events in the analysis of the study outcome. We did not analyze the temporal fluctuations of fluid overload in response to diuretics as a marker of mortality.

The study size was determined using the computer software Programs for Epidemiologists (PEPI version 4.04x). The program indicated a base of 480 patients for the 5% (α = 0.05) minimal error type I and a 20% probability error type II to estimate the OR value = 3, mortality incidence about 5% when comparing patients with the 0 to 0.5 creatinine change in relation to decreased -0.3 to 0 values, according to Lassnigg[5]. Data analysis was performed using Microsoft, Excel (Microsoft Corp, Redmond, WA, USA).

### Statistical analysis

Quantitative data were described using mean and standard deviation or the median and interquartile range in the presence of skewness. Categorical data were expressed using counts and percentages. We excluded patients who died in the trans-operative period and in the first 24 hours after surgery.

Volume accumulation and changes in creatinine were transformed to Z scores to analyze the association of unit changes in both variables. We did individual Chi-square tests to identify the potential morbidities. Due to the limited number of deaths, we analyzed the combined morbidities, including mortality, and its association with potentially predictive factors using logistic regression to obtain ORs with their respective 95% CIs.

Due to the skewness, the length of ICU stay was log-transformed. For the association of volume accumulation and length of ICU stay we used a linear regression model (r Pearson coefficient). Length of ICU stay was modeled using multiple linear regression to evaluate its relationship to a number of independent factors. In this model, changes in serum creatinine were expressed as Δ creatinine, which was calculated as: Changes in creatinine = Highest serum creatinine value - Baseline creatinine. Standardized beta coefficients were used to obtain adjusted contributions of these factors in the model. Significance level was set at α = 0.05. Data were analyzed using SPSS version 18.0.

### Results

**Figure 1:**
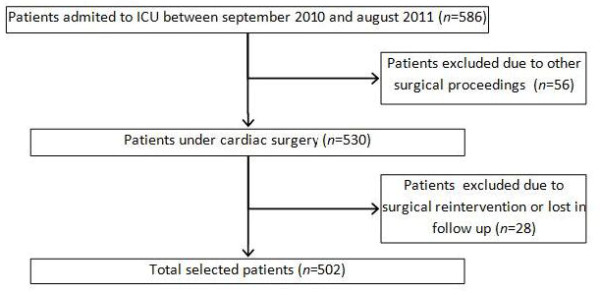
**Outline of the study**.

Five hundred and eighty-six patients were admitted to the postoperative ICU from September 2010 through September 2011. Eighty-four patients were excluded because they had undergone other surgical procedures, surgical reintervention or were lost to follow-up. Five hundred and two patients were prospectively studied (Figure 1).

### Patients Characteristics

A total of 502 patients, with a mean age of 62.4 ± 13.3 years old, 61.4% men and 38.6% women were analyzed; baseline characteristics are shown in Table 1. We also observed that only 19.6% of the patients (96) had changes in creatinine >0.3 mg/dl during their entire length of ICU stay, and only seven patients needed dialysis; 12 patients had a neurologic disorder and 30 patients had an infection.

**Table 1:** Clinical and laboratory parameters of patients obtained preoperatively

Characteristics	N	Statistic
Age, years	502	62.4±13.3
Male, nº (%)	502	308 (61.4)
Creatinine, mg/dL	502	0.9 (0.7 to 1.1)
Ejection fraction, %	319	58.9±14.3
Diabetes, nº (%)	502	122 (24.3)
Hypertension, nº (%)	502	376 (74.9)
Heart Failure, nº (%)	502	100 (19.9)
Arrhythmia, nº (%)	502	62 (12.4)
CBP time, min	500	82.9±29.5
EuroSCORE %	502	2.9 (1.6 to 5.5)
Length of ICU stay(days)	502	2.0(1.0 to 3.0)
Surgeries, nº (%)	502	
Myocardial revascularization		256 (51.0)
Valve surgery		182 (36.3)
Others		64 (12.7)

The data are presented as mean±standard deviation, median (interquartile range: P25 to P75) or count (percentage). CPB: Cardiopulmonary by-pass, EuroSCORE: ***Euro**pean **S**ystem for **C**ardiac **O**perative **R**isk **E**valuation*. ICU: Intensive Care Unit

### Death, percentage variation of fluid overload and serum changes in serum creatinine

Over the 12-month period, 17 patients died during their ICU stay in the postoperative period (3.38%). When we analyzed the association of fluid accumulation and changes in serum creatinine by Z score variables with mortality, we observed that fluid overload and changes in serum creatinine were significantly associated with mortality. For fluid overload, we found a significant increase of 1.59 in the OR (CI 1.18 to 2.14, *P *= 0.002) for death at each Z score in fluid overload. For creatinine, we found a significant increase of 2.91 in the OR for death (CI 1.92 to 4.40, *P*<0.001) at every creatinine Z score change (Table 2). We were unable to separately analyze the death event adjusted to confounding factors because the number of deaths was very small in this period of observation. Thus, we studied this relationship including combined events, such as death, infection, cardiac arrhythmia, bleeding and pulmonary edema. Logistic regression analysis of combined events and association variables revealed a significant increase in combined events when the change in serum creatinine was 0.3 to 0.6 mg/dl (OR 2.4; CI 1.24 to 4.65; *P *= 0.009), and ≥0.6 mg/dl (OR 6.17; CI 2.83 to 13.45;

**Table 2:** Association of selected variables with the occurrence of death in patients submitted to cardiac surgery.

Variables	OR	CI 95%	*P*
Z score % fluid accumulation	1.59	1.18 to 2.14	0.002
Z score change creatinine	2.91	1.92 to 4.40	<0.001

Number = 501; events = 17. OR, odds ratio; CI, confidence interval; *P*, statistical significance.

*P *<0.001). We also found a significant and independent increase in combined events when the fluid accumulation increased 10% (OR 4.43; CI: 2.08 to 9.14; *P *<0.001) (Table 3). We also calculated this model with creatinine values adjusted to volume accumulation, as described by Macedo *et al*. [19] and found similar results for changes in creatinine (OR 2.5; CI 1.31 to 4.83, *P *= 0.005 to changes of 0.3 to 0.6 mg/dL and OR 6.30; CI 2.92 to 13.58, *P *<0.001 to changes in creatinine ≥0.6 mg/dL).

**Table 3:** Association of selected variables with combined events.

Variable	Multivariable analysis		
	
	OR	CI 95%	*P*
Creatinine <0.3 mg/dL	1		<0.001
Creatinine 0.3 to 0.6 mg/dL	2.40	1.24 to 4.65	0.009
Creatinine ≥0.6 mg/dL	6.17	2.83 to13.45	<0.001
Fluid accumulation <5%	1		<0.001
Fluid accumulation 5% to 10%	1.64	0.94 to 2.84	0.078
Fluid accumulation >10%	4.43	2.08 to 9.14	<0.001
Preoperative creatinine (mg/dL)	0.93	0.60 to 1.43	0.741
Time on CPB (minutes)	1.01	0.99 to 1.01	0.268
Age (years)	0.99	0.97 to 1.02	0.898
EuroSCORE%	1.06	1.02 to 1.11	0.008

CBP, cardiopulmonary bypass; OR, odds ratio; CI, confidence interval; *P*, statistical significance. Combined events: death (n = 17), infection (n = 30), cardiac arrhythmia (n = 69), bleeding (n = 24) and pulmonary edema (n = 7).

We have to emphasize that none of the patients who died had a decreased creatinine level. Considering this observation, we did not analyze the group separately. It was possible to demonstrate that of the 157 patients who had decreased creatinine levels, only four (2.5%) presented fluid overload ≥10%.

### Length of ICU stay and percentage variation of fluid overload

**Figure 2:**
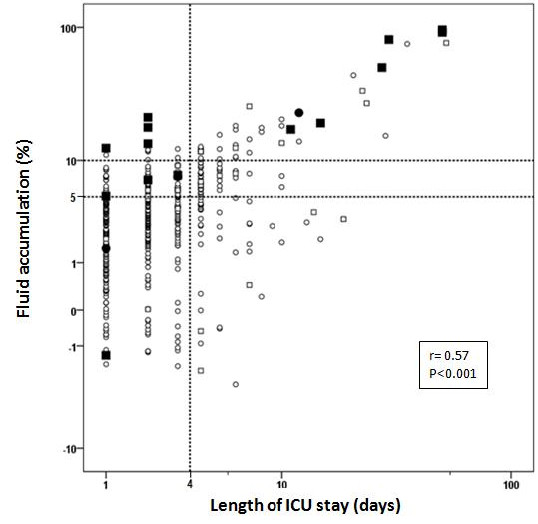
**Length of stay, fluid accumulation, changes in serum creatinine and mortality**. Black circle: non-survival with changes in serum creatinine <0.6 mg/dL; White circle: survival with changes in serum creatinine <0.6mg/dL; Black square: non-survival with changes in serum creatinine ≥0.6 mg/dL; White square: survival with changes in serum creatinine ≥0.6 mg/dL.

We found a moderate to strong magnitude relationship between the length of ICU stay and fluid overload (r = 0.57, *P *<0.001). We also found that patients who survived after four days in the ICU had no fluid accumulation (Figure 2). After analyzing the independent contribution of all parameters, we observed that a 10% fluid overload was substantially greater than 0.1 mg/dL changes in serum creatinine, in accounting for ICU stay. In this analysis, time on CPB, EuroSCORE, preoperative creatinine and age were not found to be associated with length of ICU stay (Table 4).

**Table 4:** Association of selected variables with the length of ICU stay (in log days) in patients submitted to cardiac surgery (n = 502).

Variable	Multivariable analysis		
	
	B	Beta	*P*
Δ creatinine (mg/dL)	0.01	0.11	0.003
Δ cumulative fluid (%)	0.17	0.55	<0.001
Age (years)	<0.01	0.07	0.061
Time of CPB (minutes)	<0.01	0.09	0.011
EuroSCORE%	<0.01	<0.01	0.994
Preoperative creatinine (mg/dL)	0.03	0.06	0.123

Δ(delta), variation (final value- initial value); b, angular coefficient; Beta, regression linear coefficient; CPB, cardiopulmonary bypass; n, number; *P*, statistical significance.

### Outcome of fluid overload, length of stay in ICU, change in serum creatinine and death

We found a moderate association between fluid accumulation and length of ICU stay (r = 0.57, *P *<0.001). As the majority of patients stayed only two days in the ICU, (median = 2, 1 to 3), we analyzed these patients in relation to the upper percentile (P75). We found that only 66 patients stayed more than four days in the ICU (P75). The outcome of fluid overload is time related (Figure 2). We found that ten patients died within the first four days in the ICU (P75), 71.4% with fluid accumulation ≥10%; we also found that all seven patients (100%) who died after four days in the ICU had fluid accumulation ≥10%. No patient with less than 10% fluid accumulation died during this period. When we considered the change in serum creatinine, we found that eight patients (75%) with creatinine variation ≥ 0.6mg/dL died in the first four days, and six patients (85.7%) died after a four-day stay in the ICU.

## Discussion

Our analysis describes the importance of fluid overload and changes in serum creatinine during the perioperative period following cardiac surgery as early markers of intra-ICU mortality and longer ICU stay.

To our knowledge, this study is the first to evaluate systematically fluid overload after cardiac surgery. Our most interesting finding is that the 10% fluid overload also seemed to have a significant and independent effect on the combined events, including death. This 10% fluid overload effect was observed in another study that investigated acute kidney dysfunction [17]. Sutherland *et al*., in a recent pediatric study into acute kidney dysfunction, reported a 3% increase in mortality for each 1% increase in fluid accumulation [20].

An inverse relationship between fluid accumulation and survival has been reported in several other conditions, such as in the perioperative period [21-23], acute pulmonary edema [8,24], pulmonary injury [25], sepsis [18,23,26] and acute kidney injury (AKI) [27], chronic renal failure [28,29] and decompensated heart failure [30]. When we studied the impact of all the adjusted variables on combined events, we found that the changes in serum creatinine (≥0.3 mg/dL) and fluid accumulation (≥10%) were the variables most significantly associated with mortality (Table 2). The changes in serum creatinine values and the association with mortality seems to corroborate Lassnigg's findings [5]. These results were previously observed in the perioperative period as well as in critically ill populations, because fluid overload has been associated with impaired wound healing and prolonged mechanical ventilation [31,32]. Hypervolemia also seems to be associated with cardiac edema, leading to myocyte injury [33] and malignant ventricular tachyarrhythmia [34].

We also found a significant and independent association between fluid overload and length of ICU stay, similar to those previously found in patients undergoing general surgery [35], with cardio renal syndrome and acute kidney diseases [7]. In our data, fluid overload seemed to play a much greater role than creatinine in the length of ICU stay (Table 4).

The association between classic risk factors, such as CPB [36], EuroSCORE [37] and preoperative renal dysfunction, and a higher rate of postoperative complications and longer length of hospital stay has been widely reported in the medical literature [38]. However, when we analyzed the independent contribution of variables to account for the length of ICU stay, we found that only fluid overload and changes in serum creatinine were related to length of ICU stay.

The early identification of fluid overload is essential to establish adequate management in cardiac patients, since this is regarded as the most important hemodynamic factor in the worsening of renal function in patients with congestive heart failure [39]. When cardiac dysfunction occurs, it is not unusual to find that renal function suddenly worsens [40] and that the adjustment of volume and sodium is out of control [41]. Fluid overload may be associated with myocardial edema, which, together with inflammatory changes and the activation of the renin-angiotensin system, increases the consumption of myocardial oxygen, leading to an unfavorable clinical situation [42]. It may also alter renal function due to diminished perfusion secondary to venous congestion [13,43], so reducing glomerular filtration [44].

Once renal insufficiency occurs in the setting of heart failure, the prognosis becomes poor. There is a gap in the knowledge regarding the clear distinction between pre-renal and established acute renal failure associated with decreasing compensatory mechanisms and subclinical episodes of heart and kidney dysfunction. Filling this gap is critical for the adequate management of the afflicted patients [45].

Serum creatinine levels can also undergo changes during the postoperative period after cardiac surgery due to extracorporeal circulation and hemodilution [7]. However, in our data, we found no relationship between the decreased change in creatinine and mortality, because no patient in this group died, in contrast to what was observed by Lassnigg [5].

Due the fact that only 66 patients stayed more than four days in the ICU, in our data it was impossible to detect an expressive temporal profile. However, we found none of the surviving patients had fluid overload four days after cardiac surgery. Interaction between the heart and kidneys is intense. It is also well established that renal and cardiac function are closely related to fluid control and maladjustment in one of the organs may trigger an alteration in the other [46]. Fluid accumulation may have a bidirectional association with renal dysfunction [24] and may account for this rather direct relationship between heart and kidney, the cardio renal syndrome [24]. In order to establish adequate cross-talk between them, it is necessary to maintain an adequate blood volume and hemodynamic stability [30]. This is so important that several authors have identified fluid overload as a new biomarker of dysfunction of the cardio renal syndrome [41].

### Study limitations

The present study has some limitations. This is a prospective and observational study, prone to bias of selection and residual confounding. Our population included patients with and without sepsis, the number of deaths was small (17 patients), and few patients required dialysis (seven patients). Also, the percentage time of the fluid overload was not considered in this study, a factor which, as is known, may interfere in the length of hospitalization and mortality [15]. Therefore, it is also difficult to distinguish precisely whether the excess of fluid balance is the cause or the result of postoperative complications. It also remains unclear whether fluid restriction or the use of diuretics after cardiac surgery reduces morbidity and mortality [23].

We did not analyze the mechanisms involved in the development of fluid overload, including the contribution of volume infusion of fluids, the particular type of fluid administered or the absence of response to diuretics. These points need to be addressed in further studies.

Other measurements associated with fluid overload, such as central venous pressure or pulmonary capillary pressure, and pro BNP measures could be performed. In fact, these parameters were obtained from several patients, but not in a systematic protocol. Therefore, we did not analyze and present the related data. However, our results strongly indicate that fluid accumulation control and changes in serum creatinine together may represent valuable tools to detect a population with a higher cardiovascular risk among patients treated in a 'real life' scenario.

## Conclusions

In summary, we showed a significant and independent association between fluid overload and changes in serum creatinine in relation to combined events of death, infection, bleeding, arrhythmia and pulmonary edema in postoperative cardiac surgery. We also found that fluid overload was the variable most related to length of stay in postoperative care following cardiac surgery.

As such, our findings contribute towards expanding the knowledge regarding this still unclear field of the functional interaction between the kidneys and the heart. Further randomized and controlled studies are needed to define whether early fluid overload detection would be an unrecognized form of initial renal failure and a target treatment to lower mortality associated with cardiac surgery.

Fluid therapy is widely endorsed for the resuscitation of critically ill patients across a range of conditions. Yet, the approach to fluid therapy is subject to substantial variation in clinical practice. Emerging evidence shows that choice, timing and amount of fluid therapy affect outcome. Further studies would need to focus on these aspects of fluid therapy by means of larger, more rigorous and blinded controlled trials.

## Key messages

• Fluid overload is independently associated with combined events in post operative cardiac surgery.

• Increase in serum creatinine is independently associated with combined events in post operative cardiac surgery.

• Fluid overload is substantially more effective in accounting for ICU stay than 0.1mg/dL changes in serum creatinine.

• The early identification of fluid overload is essential to establish adequate management in cardiac patients.

## Abbreviations

AKI: acute kidney injury; b: angular coefficient obtained in a multiple linear regression model; CI: confidence interval; CPB: cardiopulmonary bypass; Δ variation: (final value - initial value); EuroSCORE: European System for Cardiac Operative Risk*; *OR: odds ratio; P25 to P75: interquartile range.

## Competing interests

The authors declare that they have no competing interests.

## Authors' contributions

AS and MCI conceived the study, participated in study design, prepared data, performed statistical analysis and wrote the paper. LVS, CRB, WRM and JRV participated in study design, prepared data, collected data and wrote the paper. EMCP, RE, LA and FCC contributed in the writing and critical appraisal of the manuscript. All authors read and approved the final manuscript.

## References

[B1] Hoste EA Kellum JA Katz NM Rosner MH Haase M Ronco C Epidemiology of acute kidney injury Contrib Nephrol 2010 165 1 8 20427949 10.1159/000313737

[B2] Mangano CM Diamondstone LS Ramsay JG Aggarwal A Herskowitz A Mangano DT Renal dysfunction after myocardial revascularization: risk factors, adverse outcomes, and hospital resource utilization. The Multicenter Study of Perioperative Ischemia Research Group Ann Intern Med 1998 128 194 203 9454527 10.7326/0003-4819-128-3-199802010-00005

[B3] Chertow GM Lazarus JM Christiansen CL Cook EF Hammermeister KE Grover F Daley J Preoperative renal risk stratification Circulation 1997 95 878 884 10.1161/01.CIR.95.4.878 9054745

[B4] Loef BG Epema AH Smilde TD Henning RH Ebels T Navis G Stegeman CA Immediate postoperative renal function deterioration in cardiac surgical patients predicts in-hospital mortality and long-term survival J Am Soc Nephrol 2005 16 195 200 15563558 10.1681/ASN.2003100875

[B5] Lassnigg A Schmidlin D Mouhieddine M Bachmann LM Druml W Bauer P Hiesmayr M Minimal changes of serum creatinine predict prognosis in patients after cardiothoracic surgery: a prospective cohort study J Am Soc Nephrol 2004 15 1597 1605 10.1097/01.ASN.0000130340.93930.DD 15153571

[B6] Cotter G Metra M Milo-Cotter O Dittrich HC Gheorghiade M Fluid overload in acute heart failure--re-distribution and other mechanisms beyond fluid accumulation Eur J Heart Fail 2008 10 165 169 10.1016/j.ejheart.2008.01.007 18279771

[B7] Cruz DN Soni S Slavin L Ronco C Maisel A Biomarkers of cardiac and kidney dysfunction in cardiorenal syndromes Contrib Nephrol 2010 165 83 92 20427958 10.1159/000313747

[B8] Bagshaw SM Cruz DN Fluid overload as a biomarker of heart failure and acute kidney injury Contrib Nephrol 2010 164 54 68 20427994 10.1159/000313721

[B9] Hillege HL Girbes AR de Kam PJ Boomsma F de Zeeuw D Charlesworth A Hampton JR van Veldhuisen DJ Renal function, neurohormonal activation, and survival in patients with chronic heart failure Circulation 2000 102 203 210 10.1161/01.CIR.102.2.203 10889132

[B10] Bagshaw SM Brophy PD Cruz D Ronco C Fluid balance as a biomarker: impact of fluid overload on outcome in critically ill patients with acute kidney injury Critical Care 2008 12 169 10.1186/cc6948 18671831 PMC2575565

[B11] Schrier RW Water and sodium retention in edematous disorders: role of vasopressin and aldosterone Am J Med 2006 119 S47 53 10.1016/j.amjmed.2006.05.007 16843085

[B12] Gheorghiade M Follath F Ponikowski P Barsuk JH Blair JE Cleland JG Dickstein K Drazner MH Fonarow GC Jaarsma T Jondeau G Sendon JL Mebazaa A Metra M Nieminen M Pang PS Seferovic P Stevenson LW van Veldhuisen DJ Zannad F Anker SD Rhodes A McMurray JJ Filippatos G European Society of Cardiology; European Society of Intensive Care Medicine Assessing and grading congestion in acute heart failure: a scientific statement from the Acute Heart Failure Committee of the Heart Failure Association of the European Society of Cardiology and endorsed by the European Society of Intensive Care Medicine Eur J Heart Fail 2010 12 423 433 10.1093/eurjhf/hfq045 20354029

[B13] Soni SS Ronco C Katz N Cruz DN Early diagnosis of acute kidney injury: the promise of novel biomarkers Blood Purif 2009 28 165 174 10.1159/000227785 19590184

[B14] Iwanaga Y Miyazaki S Heart failure, chronic kidney disease, and biomarkers--an integrated viewpoint Circ J 2010 74 1274 1282 10.1253/circj.CJ-10-0444 20558890

[B15] Bouchard J Soroko SB Chertow GM Himmelfarb J Ikizler TA Paganini EP Mehta RL Fluid accumulation, survival and recovery of kidney function in critically ill patients with acute kidney injury Kidney Int 2009 76 422 427 10.1038/ki.2009.159 19436332

[B16] Goldstein SL Currier H Graf C Cosio CC Brewer ED Sachdeva R Outcome in children receiving continuous venovenous hemofiltration Pediatrics 2001 107 1309 1312 10.1542/peds.107.6.1309 11389248

[B17] Gillespie RS Seidel K Symons JM Effect of fluid overload and dose of replacement fluid on survival in hemofiltration Pediatr Nephrol 2004 19 1394 1399 10.1007/s00467-004-1655-1 15517417

[B18] Bouchard J Mehta RL Fluid balance issues in the critically ill patient Contrib Nephrol 2010 164 69 78 20427995 10.1159/000313722

[B19] Macedo E Bouchard J Soroko SH Chertow GM Himmelfarb J Ikizler TA Paganini EP Mehta RL Fluid accumulation, recognition and staging of acute kidney injury in critically-ill patients Critical Care 2010 14 R82 10.1186/cc9004 20459609 PMC2911707

[B20] Sutherland SM Zappitelli M Alexander SR Chua AN Brophy PD Bunchman TE Hackbarth R Somers MJ Baum M Symons JM Flores FX Benfield M Askenazi D Chand D Fortenberry JD Mahan JD McBryde K Blowey D Goldstein SL Fluid overload and mortality in children receiving continuous renal replacement therapy: the prospective pediatric continuous renal replacement therapy registry Am J Kidney Dis 2010 55 316 325 10.1053/j.ajkd.2009.10.048 20042260

[B21] Brandstrup B Tonnesen H Beier-Holgersen R Hjortso E Ording H Lindorff-Larsen K Rasmussen MS Lanng C Wallin L Iversen LH Gramkow CS Okholm M Blemmer T Svendsen PE Rottensten HH Thage B Riis J Jeppesen IS Teilum D Christensen AM Graungaard B Pott F Danish Study Group on Perioperative Fluid Therapy Effects of intravenous fluid restriction on postoperative complications: comparison of two perioperative fluid regimens: a randomized assessor-blinded multicenter trial Ann Surg 2003 238 641 648 10.1097/01.sla.0000094387.50865.23 14578723 PMC1356139

[B22] Kleespies A Thiel M Jauch KW Hartl WH Perioperative fluid retention and clinical outcome in elective, high-risk colorectal surgery Int J Colorectal Dis 2009 24 699 709 10.1007/s00384-009-0659-5 19221767

[B23] Wei S Tian J Song X Chen Y Association of perioperative fluid balance and adverse surgical outcomes in esophageal cancer and esophagogastric junction cancer Ann Thorac Surg 2008 86 266 272 10.1016/j.athoracsur.2008.03.017 18573435

[B24] Ronco C Maisel A Volume overload and cardiorenal syndromes Congest Heart Fail 2010 16 Suppl 1 Si iv quiz Svi 20653717 10.1111/j.1751-7133.2010.00176.x

[B25] Sakr Y Vincent JL Reinhart K Groeneveld J Michalopoulos A Sprung CL Artigas A Ranieri VM High tidal volume and positive fluid balance are associated with worse outcome in acute lung injury Chest 2005 128 3098 3108 10.1378/chest.128.5.3098 16304249

[B26] Hilton AK Bellomo R Totem and taboo: fluids in sepsis Crit Care 2011 15 164 10.1186/cc10247 21672278 PMC3218999

[B27] Selewski DT Cornell TT Lombel RM Blatt NB Han YY Mottes T Kommareddi M Kershaw DB Shanley TP Heung M Weight-based determination of fluid overload status and mortality in pediatric intensive care unit patients requiring continuous renal replacement therapy Intensive Care Med 2011 37 1166 1173 10.1007/s00134-011-2231-3 21533569 PMC3315181

[B28] Uchino S Bellomo R Kellum JA Morimatsu H Morgera S Schetz MR Tan I Bouman C Macedo E Gibney N Tolwani A Oudemans-Van Straaten HM Ronco C Beginning and Ending Supportive Therapy for the Kidney (B.E.S.T. Kidney) Investigators Writing Committee Patient and kidney survival by dialysis modality in critically ill patients with acute kidney injury Int J Artif Organs 2007 30 281 292 17520564 10.1177/039139880703000402

[B29] Bonello M House AA Cruz D Asuman Y Andrikos E Petras D Strazzabosco M Ronco F Brendolan A Crepaldi C Nalesso F Ronco C Integration of blood volume, blood pressure, heart rate and bioimpedance monitoring for the achievement of optimal dry body weight during chronic hemodialysis Int J Artif Organs 2007 30 1098 1108 18203072 10.1177/039139880703001210

[B30] Damman K Voors AA Hillege HL Navis G Lechat P van Veldhuisen DJ Dargie HJ Congestion in chronic systolic heart failure is related to renal dysfunction and increased mortality Eur J Heart Fail 2010 12 974 982 10.1093/eurjhf/hfq118 20685688

[B31] Bundgaard-Nielsen M Secher NH Kehlet H 'Liberal' vs. 'restrictive' perioperative fluid therapy--a critical assessment of the evidence Acta Anaesthesiol Scand 2009 53 843 851 10.1111/j.1399-6576.2009.02029.x 19519723

[B32] Wiedemann HP Wheeler AP Bernard GR Thompson BT Hayden D deBoisblanc B Connors AF Jr Hite RD Harabin AL Comparison of two fluid-management strategies in acute lung injury N Engl J Med 2006 354 2564 2575 16714767 10.1056/NEJMoa062200

[B33] Peacock WF 4th De Marco T Fonarow GC Diercks D Wynne J Apple FS Wu AH Cardiac troponin and outcome in acute heart failure N Engl J Med 2008 358 2117 2126 10.1056/NEJMoa0706824 18480204

[B34] Ip JE Cheung JW Park D Hellawell JL Stein KM Iwai S Liu CF Lerman BB Markowitz SM Temporal associations between thoracic volume overload and malignant ventricular arrhythmias: a study of intrathoracic impedance J Cardiovasc Electrophysiol 2011 22 293 299 10.1111/j.1540-8167.2010.01924.x 20946226

[B35] Cooperman LH Price HL Pulmonary edema in the operative and postoperative period: a review of 40 cases Ann Surg 1970 172 883 891 10.1097/00000658-197011000-00014 5477659 PMC1397352

[B36] Swaminathan M Phillips-Bute BG Conlon PJ Smith PK Newman MF Stafford-Smith M The association of lowest hematocrit during cardiopulmonary bypass with acute renal injury after coronary artery bypass surgery Ann Thorac Surg 2003 76 784 791 discussion 792 10.1016/S0003-4975(03)00558-7 12963200

[B37] Roques F Nashef SA Michel P Gauducheau E de Vincentiis C Baudet E Cortina J David M Faichney A Gabrielle F Gams E Harjula A Jones MT Pintor PP Salamon R Thulin L Risk factors and outcome in European cardiac surgery: analysis of the EuroSCORE multinational database of 19030 patients Eur J Cardiothorac Surg 1999 15 816 822 discussion 822-823 10.1016/S1010-7940(99)00106-2 10431864

[B38] Chertow GM Burdick E Honour M Bonventre JV Bates DW Acute kidney injury, mortality, length of stay, and costs in hospitalized patients J Am Soc Nephrol 2005 16 3365 3370 10.1681/ASN.2004090740 16177006

[B39] Ponikowski P Ronco C Anker SD Cardiorenal syndromes--recommendations from clinical practice guidelines: the cardiologist's view Contrib Nephrol 2010 165 145 152 20427964 10.1159/000313753

[B40] Metra M Nodari S Parrinello G Bordonali T Bugatti S Danesi R Fontanella B Lombardi C Milani P Verzura G Cotter G Dittrich H Massie BM Dei Cas L Worsening renal function in patients hospitalised for acute heart failure: clinical implications and prognostic significance Eur J Heart Fail 2008 10 188 195 10.1016/j.ejheart.2008.01.011 18279773

[B41] Mebazaa A Congestion and cardiorenal syndromes Contrib Nephrol 2010 165 140 144 20427963 10.1159/000313752

[B42] Jessup M Costanzo MR The cardiorenal syndrome: do we need a change of strategy or a change of tactics? J Am Coll Cardiol 2009 53 597 599 10.1016/j.jacc.2008.11.012 19215834

[B43] Amann K Wanner C Ritz E Cross-talk between the kidney and the cardiovascular system J Am Soc Nephrol 2006 17 2112 2119 10.1681/ASN.2006030204 16825329

[B44] Ljungman S Laragh JH Cody RJ Role of the kidney in congestive heart failure. Relationship of cardiac index to kidney function Drugs 1990 39 10 21 10.2165/00003495-199000394-00004 2354670

[B45] Macedo E Mehta R Prerenal azotemia in congestive heart failure Contrib Nephrol 2010 164 79 87 20427996 10.1159/000313723

[B46] Ronco C Cardiorenal syndromes: definition and classification Contrib Nephrol 2010 164 33 38 20427991 10.1159/000313718

